# Microbial predation accelerates granulation and modulates microbial community composition

**DOI:** 10.1186/s12866-021-02156-8

**Published:** 2021-03-27

**Authors:** Siew Herng Chan, Muhammad Hafiz Ismail, Chuan Hao Tan, Scott A. Rice, Diane McDougald

**Affiliations:** 1 Singapore Centre for Environmental Life Sciences Engineering, Nanyang Technological University, 60 Nanyang Drive, Singapore, 637551 Singapore; 2 Interdisciplinary Graduate School, Nanyang Technological University, Singapore, Singapore; 3 School of Biological Sciences, Nanyang Technological University, Singapore, Singapore; 4 School of Materials Science and Engineering, Nanyang Technological University, Singapore, Singapore; 5 The iThree Institute, University of Technology Sydney, Sydney, Australia

**Keywords:** Granulation, Protozoa, Bacteriophage, Metagenomics, Activated sludge, Microbial predation

## Abstract

**Background:**

Bacterial communities are responsible for biological nutrient removal and flocculation in engineered systems such as activated floccular sludge. Predators such as bacteriophage and protozoa exert significant predation pressure and cause bacterial mortality within these communities. However, the roles of bacteriophage and protozoan predation in impacting granulation process remain limited. Recent studies hypothesised that protozoa, particularly sessile ciliates, could have an important role in granulation as these ciliates were often observed in high abundance on surfaces of granules. Bacteriophages were hypothesized to contribute to granular stability through bacteriophage-mediated extracellular DNA release by lysing bacterial cells. This current study investigated the bacteriophage and protozoan communities throughout the granulation process. In addition, the importance of protozoan predation during granulation was also determined through chemical killing of protozoa in the floccular sludge.

**Results:**

Four independent bioreactors seeded with activated floccular sludge were operated for aerobic granulation for 11 weeks. Changes in the phage, protozoa and bacterial communities were characterized throughout the granulation process. The filamentous phage, Inoviridae, increased in abundance at the initiation phase of granulation. However, the abundance shifted towards lytic phages during the maturation phase. In contrast, the abundance and diversity of protozoa decreased initially, possibly due to the reduction in settling time and subsequent washout. Upon the formation of granules, ciliated protozoa from the class *Oligohymenophorea* were the dominant group of protozoa based on metacommunity analysis. These protozoa had a strong, positive-correlation with the initial formation of compact aggregates prior to granule development. Furthermore, chemical inhibition of these ciliates in the floccular sludge delayed the initiation of granule formation. Analysis of the bacterial communities in the thiram treated sludge demonstrated that the recovery of ‘*Candidatus* Accumulibacter’ was positively correlated with the formation of compact aggregates and granules.

**Conclusion:**

Predation by bacteriophage and protozoa were positively correlated with the formation of aerobic granules. Increases in Inoviridae abundance suggested that filamentous phages may promote the structural formation of granules. Initiation of granules formation was delayed due to an absence of protozoa after chemical treatment. The presence of ‘*Candidatus* Accumulibacter’ was necessary for the formation of granules in the absence of protozoa.

**Supplementary Information:**

The online version contains supplementary material available at 10.1186/s12866-021-02156-8.

## Background

Aerobic granular sludge is a complex, human engineered ecosystem consisting of highly diverse and functional microbial communities that are utilized for specific biological functions [1, 2]. These densely packed biofilm aggregates are typically developed from activated floccular sludge. Using laboratory sequencing batch reactors (SBRs), the formation of aerobic granules from flocs has been improved with the concomitant increased understanding of the effects of operating conditions such as hydrodynamic shear force, settling time, hydraulic retention time and discharging time [3–7].

In contrast to the impact of physical factors, the biological processes that drive granule formation are less well understood. For example, N-acyl-homoserine-lactone (AHL) mediated quorum sensing was found to positively correlate with the formation of granules from floccular sludge [8]. Furthermore, the addition of AHLs to the SBR markedly increased the production of EPS, which mediates contact between bacterial cells [8, 9]. Other biological factors such as predation have been demonstrated to enhance biofilm formation for several bacterial species [10–12]. Predation on those free-living bacteria may therefore represent a strong pressure selecting for bacteria that are tightly embedded in aggregates of biomass. Bacteriophages are highly abundant in engineered wastewater systems, appear to be active components of activated sludge systems and are able to infect both planktonic and biofilm associated bacterial cells [13–15].

Phage-mediated mortality has the potential to influence the treatment performance of a system through controlling the abundance of key functional groups, leading to their utilisation as a biocontrol strategy to lyse filamentous bacteria that are responsible for bulking in activated sludge [16, 17]. In contrast, bacteriophage predation in wastewater systems has been demonstrated to cause the collapse of reactors [18] and the failure of bacterial biological processes such as phosphorus removal and nitrification [18, 19]. Bacteriophage predation has recently been suggested to mediate the release of extracellular DNA via the lysis of bacterial cells, which plays a role in providing structural stability to granules [20].

Protozoa are abundant in activated floccular sludge systems and play an important role in the predation of suspended bacteria, which aids in the clarification of wastewater effluent [21]. In addition, previous studies of aerobic granulation systems demonstrated an abundance of sessile ciliates on the surface of aerobic granules [21–24]. Electron microscopy of granular surfaces revealed the attachment of bacteria on the stalks of sessile ciliates [24]. Weber et al. [24] further hypothesised that these sessile ciliates may act as nucleating agents for the attachment of bacteria. Taken together, these studies strongly suggest that protozoan predation may have a role in promoting aerobic granulation. However, the role of protozoa in the formation of aerobic granules remains unclear to date.

Here, bacteriophage, protozoan and bacterial communities were characterized throughout the aerobic granulation process. The dynamics of different bacteriophage families were investigated to elucidate their role in granulation. Additionally, the succession of protozoan communities was tracked during the aerobic granulation process and the inhibition of protozoa was performed to determine the potential role of protozoan predation in driving aerobic granulation. It is hypothesised that protozoan predation can promote the formation of granules through grazing selection pressure and/or via a structural role. Microbial community analysis indicated that there was an increase in the abundance of non-lytic, filamentous Inoviridae bacteriophages during the initiation phase of granulation when compact aggregates were formed. In addition, the abundance and diversity of protozoa decreased significantly during the aerobic granulation process. Our results also demonstrated that the absence of protozoa did not negatively affect the formation of mature granules, although there was a delay in the formation of compact aggregates in the absence of protozoa.

## Results

### Development and microscopic observations of aerobic granular sludge

**Fig. 1:**
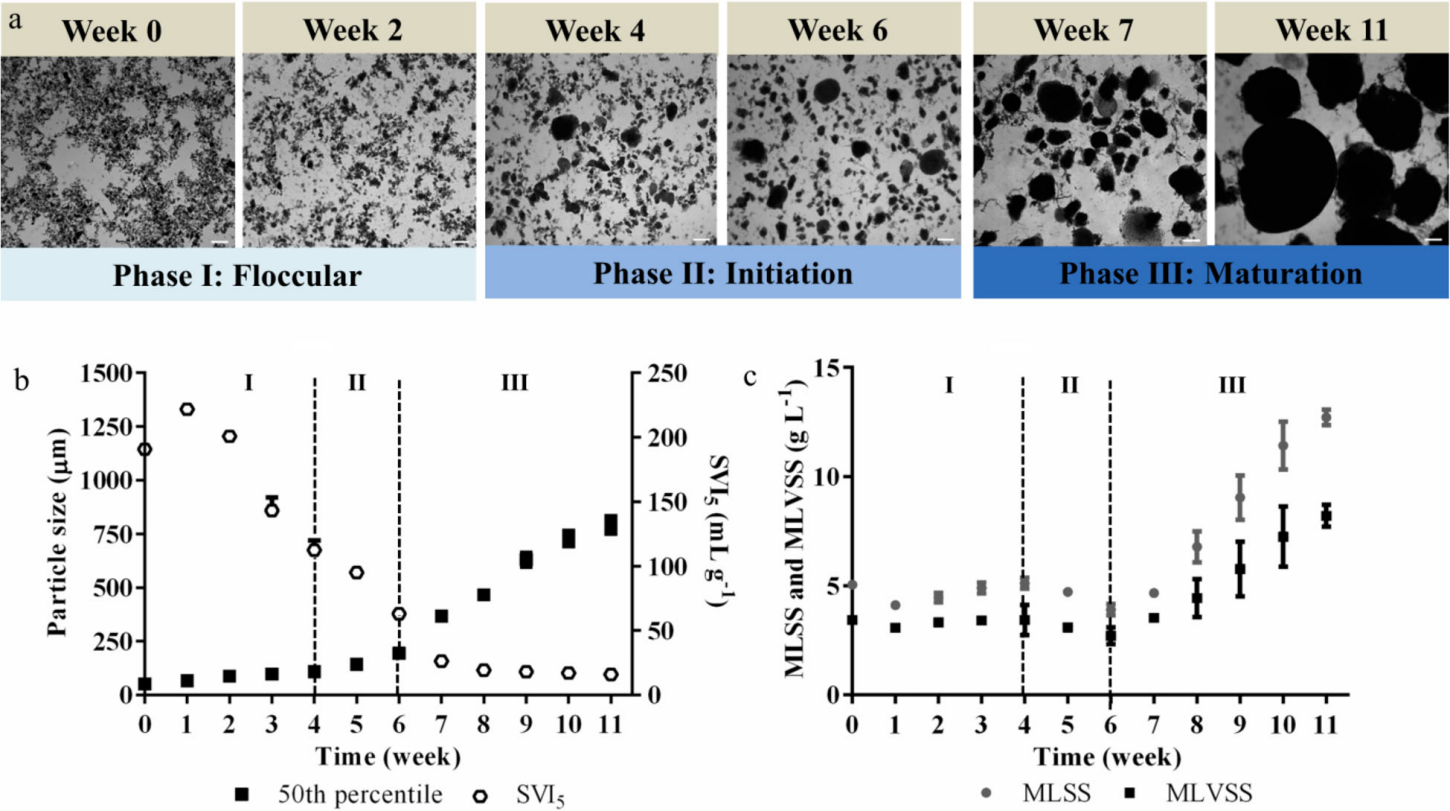
Development of granules from floccular sludge. **a** Development of small granules from floccular sludge over 11 weeks based on microscopic visualizations of sludge samples. **b** Average particle size distribution and SVI_5_ in 4 SBRs and 50th percentile (filled square) represent the percentage of total particles below the corresponding size distribution and the compactness of sludge particles as measured by SVI_5_ (open circle), respectively. **c** Average sludge biomass concentrations represented by both MLSS (filled circle) and MLVSS (filled squares). Error bars represent standard deviations (*n* = 4). Magnification × 40 (Bar, 100 μm)

Activated floccular sludge was used to seed the SBRs, which were operated under conditions optimal for the aerobic granulation process over a period of 11 weeks. The granulation process has five distinct phases: floccular, initiation, maturation, maintenance and dispersal [8]. Here, only three phases of floccular, initiation and maturation phases were observed (Fig. 1a).

During Phase I, the floccular biomass had a mean particle size of 51.3 ± 2.2 μm (50th percentile) (Fig. 1b). Aerobic granules are typically defined as dense and compact aggregates characterized by a minimum particle size of 100 μm and a SVI_5_ of 50 mL g^− 1^ or less [25]. Initial decreases in settling time from 120 to 56 min resulted in a 10.5% average loss of biomass (MLSS decreased from 5.0 ± 0.1 to 4.1 ± 0.1 g L^− 1^) by the end of week 1 (Fig. 1c). The SVI_5_ of the floccular sludge increased from 190.8 ± 2.0 to 221.8 ± 5.4 mL g^− 1^, which indicated poor settling of the floccular sludge (Fig. 1b).

By Phase II, compact aggregates were observed in the floccular sludge at week 4 and the mean particle size was 96.2 μm (50th percentile) (Fig. 1b). Subsequent decreases in settling time from 56 to 24 min did not result in a decrease in overall biomass until week 4 (MLSS increased from 4.9 ± 0.4 to 5.1 ± 0.4 g L^− 1^) when the sludge biomass entered the Phase II. During weeks 4 to 6 of Phase II, the settling time was reduced from 24 to 5 min, which resulted in an average of 23.7% loss of biomass (MLSS decreased from 5.1 ± 0.4 to 3.9 ± 0.5 g L^− 1^) (Fig. 1b). This reduction in settling time also coincided with an increase in mean particle size from 108.5 ± 6.9 to 193.0 ± 16.7 μm (50th percentile) (Fig. 1b). In addition, the SVI_5_ also decreased 44% from 112.5 ± 13.2 to 63.0 ± 6.5 mL g^− 1^ (Fig. 1c).

By week 7, the sludge biomass had entered Phase III of the aerobic granulation process. The mean particle size of the sludge biomass increased 90% from 193.0 ± 16.7 μm in week 6 to 367.0 ± 68.1 μm in week 7 (50th percentile) (Fig. 1b). The particle size and SVI_5_ of the sludge biomass continued to increase and decrease, respectively, over the remaining weeks. The MLSS of the sludge steadily increased from week 7 onwards (Fig. 1c). Over the entire 11 weeks, the reduction in settling time from 120 to 5 min was linked to the appearance of high density and compact sludge particles. This was associated with a mean particle size increase from 51.3 ± 2.2 to 792.4 ± 130.6 μm (Fig. 1b). Similarly, the SVI_5_ decreased significantly from 190.8 ± 2.5 to 16.0 ± 2.1 mL g^− 1^ (Fig. 1b). In addition, the MLSS of the sludge also increased from 3.9 ± 0.5 in week 6 to 12.7 ± 0.6 mL g^− 1^ by the end of week 11. These observations indicated that the sludge biomass was mostly in granular form.

### Microbial community composition of floccular and granular sludge

Here, the total genomic DNA of the granular sludge was sequenced to track the diversity and changes in bacteria abundance as granulation takes place over 11 weeks of reactor operation. Clustering based on the relative abundance of the microbial communities suggested that in the early floccular stages (weeks 0 and 1), the communities were similar across the 4 SBRs (Fig. S1a). However, from week 2, the communities between the reactors diverged, as reflected in changes in the community composition, as the reactors underwent granulation. Despite this, PERMANOVA showed that the reactors are not statistically different from each other (*P* = 0.184) (Table S1).

**Fig. 2:**
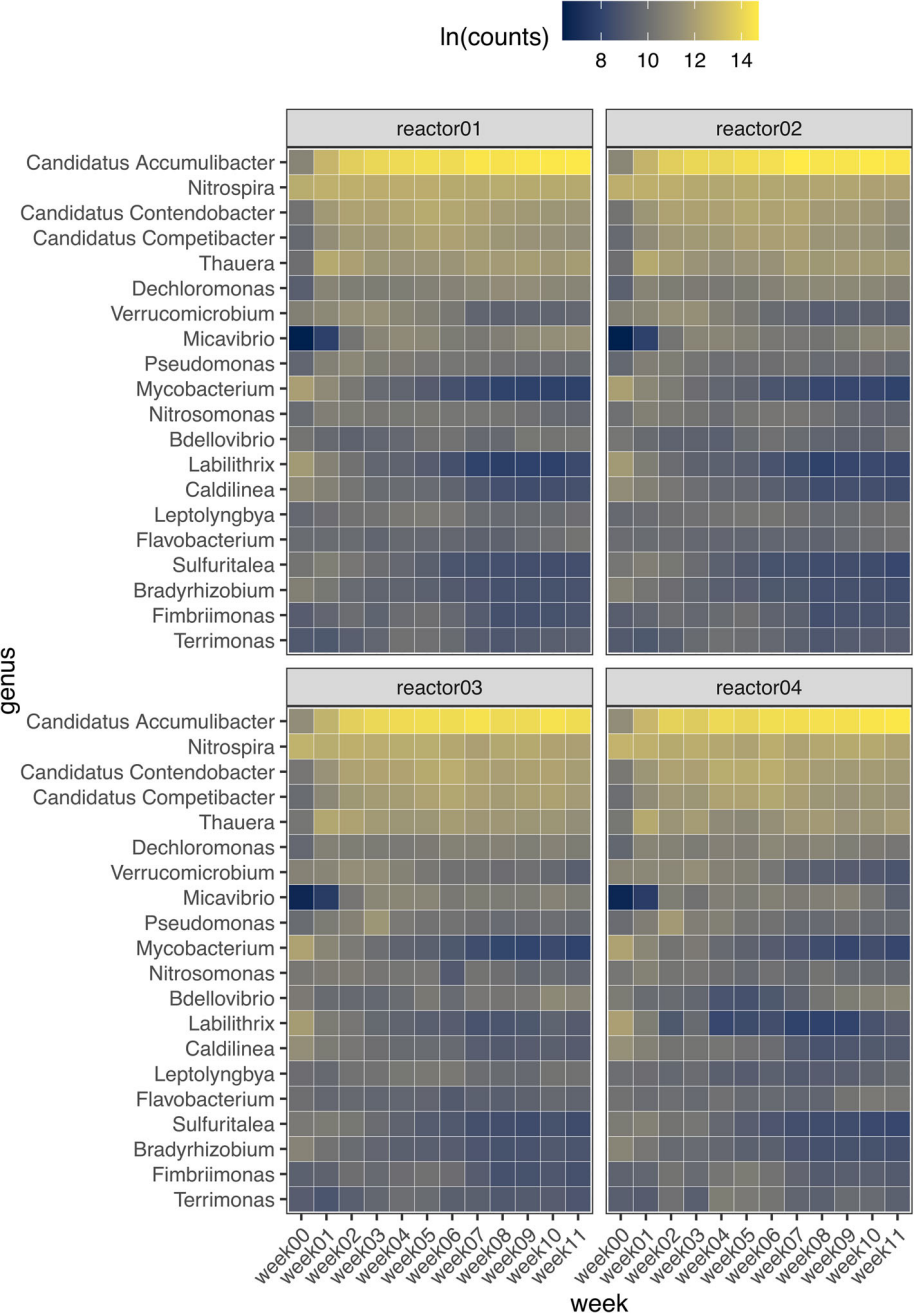
Total abundance of bacterial populations in 4 SBRs over 11 weeks of granulation. The top 20 abundant bacterial genus based on metagenome (DNA) reads. All read counts are natural log transformed before analysis

The genus ‘*Candidatus* Accumulibacter’, which is a polyphosphate accumulating organism (PAO) and nitrifier from the phylum Proteobacteria, was the most abundant, with an average increase from 3.6 to 63.53% by week 11 (Fig. 2). ‘*Candidatus* Competibacter’ and ‘*Candidatus* Contendobacter’, glycogen accumulating organisms (GAOs), did not change appreciably in abundance, between 0.97 to 3.11% and 1.47 to 3.8%, respectively (Fig. 2). Nitrifiers, such as *Nitrospira*, progressively decreased from 16.45 to 6.06% over the course of the experiment. There was a peak of *Thauera* (a denitrifier) at week 1 at 10.98% but reduced to 3.88% by the end of the experiment. The other members of the top 20 genera generally had a lower abundance with *Terrimonas* at the lowest between 0.3 and 0.97% (Fig. 2).

**Fig. 3:**
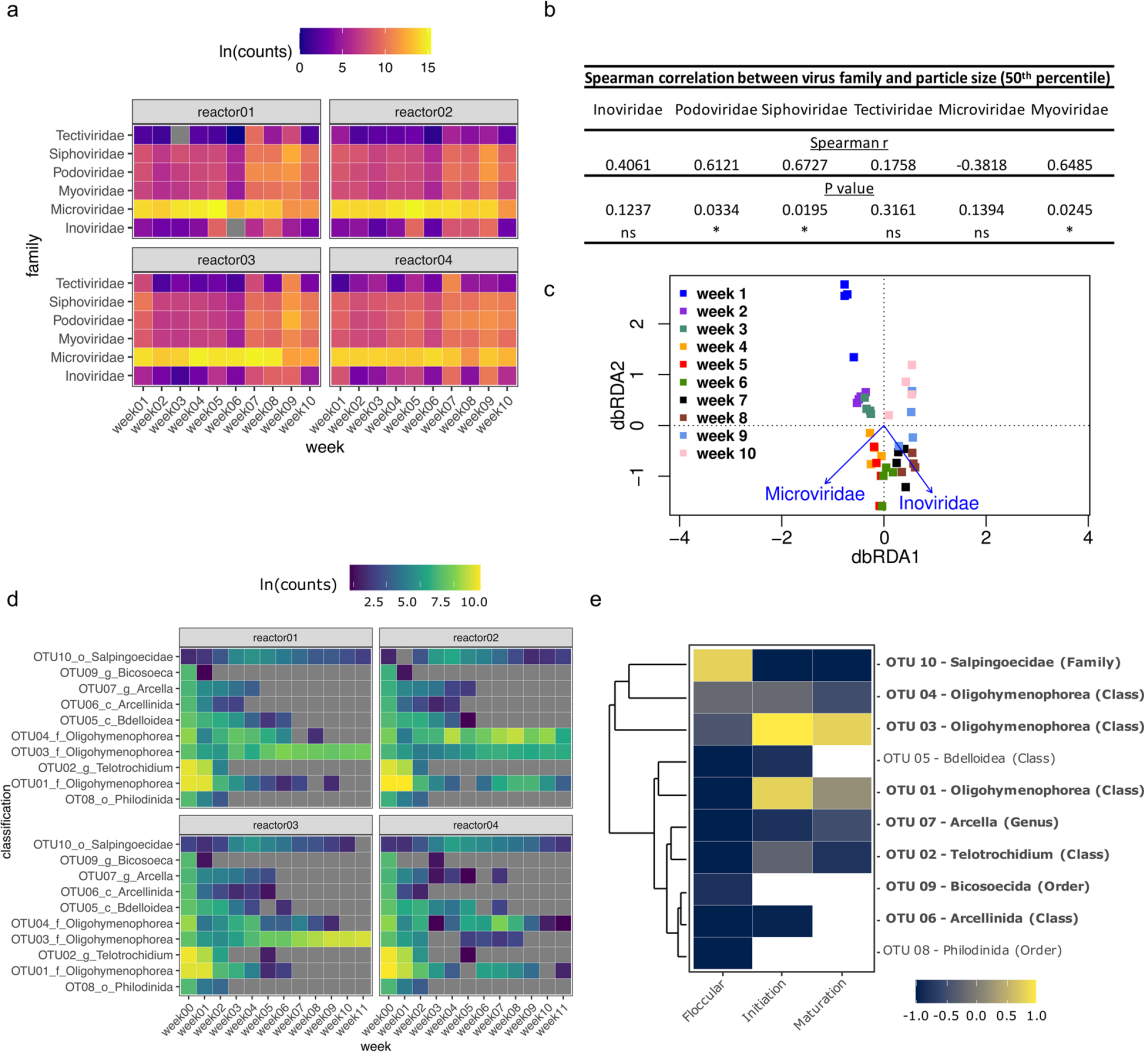
Total abundance of predator populations in 4 SBRs over 11 weeks of granulation. **a** Viral sequences were classified accordingly to family level (**b**) with corresponding Spearman correlation between viral families and 50th percentile sludge particle size. **c** Distance based redundancy analysis (dbRDA) ordination of the effect of viral dynamics on the changes in bacterial community with aerobic granulation. **d** Top 10 most abundant eukaryotic OTUs with (**e**) corresponding Spearman correlation with 50th percentile sludge particle size differentiated into the three phases of granulation. Protozoan OTUs are in bold. All read counts are natural log transformed before analysis. Viral analyses were based on viral metagenome (DNA) reads while eukaryotic OTUs were based on Ribotagger tags (RNA). The correlation matrixes were clustered based on Bray Curtis distance with between-group average linkage (UPGMA) method. The correlation matrixes are color-coded where yellow and blue indicate positive and negative correlations, respectively. Where correlations cannot be made, the tile is coloured white

Bacteriophages exert a complex influence over their microbial hosts and additionally may play a structural component of the matrix [26–28]. Therefore, the relationship between granulation and bacteriophage community dynamics were also investigated here. Only DNA bacteriophages were targeted here and their sequences were assembled into viral contigs to study their relative abundance during granulation (Fig. 3a). Microviridae were the most abundant and present in all samples throughout reactor operation at 17 to 99.9%. At the end of the initiation phase (week 7), Podoviridae and Siphoviridae began to significantly increase in abundance and at week 9, were the most abundant viral families after Microviridae at 11.15% ± 1.66 and 8.34% ± 0.96%, respectively. Inoviridae had an increase in abundance to 0.2% ± 0.05% when the sludge developed into compact aggregates (week 5) and peaked at week 9 at 1.46% ± 0.32% (Fig. 3a). There was a positive correlation between the increasing granule particle size and the viral counts of Siphoviridae, Microviridae and Myoviridae (Fig. 3b). Additionally, a distance based redundancy analysis (dbRDA) was performed to identify covariates which have an effect on the changes in bacterial community using viral family abundance [29]. This analysis suggested the Microviridae and Inoviridae viral families had an effect on the changes in bacterial community composition during the initiation phase (weeks 4 to 7) and maturation phase (weeks 8 to 9), respectively (Fig. 3c).

The effect of protozoan predation on aerobic granulation was investigated via total RNA sequencing as metagenomic sequencing did not yield sufficient reads for classification and annotation of eukaryotic sequences beyond the class level (Fig. S2). The abundance of the microbial populations was represented by the number of sequencing reads detected per OTU. Mean values were calculated for the number of sequences per OTU to represent the abundance in the four SBRs. A total of 10 OTUs represented approximately 95% of all sequencing reads. Within these 10 OTUs, there were 8 protozoan OTUs which were mostly represented by the genus *Telotrochidium* (OTU02)*,* class *Oligohymenophorea* (OTU01, 03 and 04), genus *Arcella* and order *Salpingoecidae* (Fig. 3d).

The genus *Telotrochidium* is a group of free swimming peritrichous ciliates while the genus *Arcella,* and the family of *Salpingoecidae* represent testate amoebae and flagellates, respectively (Fig. 3d). The class *Oligohymenophorea* represents a large class of ciliated protozoa. Both OTU05 and 08 represented rotifers, which are metazoan predators of suspended microorganisms (Fig. 3d). During Phase I, the abundance of *Telotrochidium* (OTU02) decreased sharply by week 2 and was not detected in most reactors in the following weeks. The family *Oligohymenophorea* OTU01 also demonstrated gradual decline in abundance from week 0 to 3. Both *Oligohymenophorea* (OTU 03 and 04) were constantly detected during Phase I in all reactors except in reactor 4, where it was absent at week 03. *Salpingoecidae* (OTU10), of the flagellate family, was also constantly present from Phase I to III. However, as compact aggregates and granules formed by Phase II and III respectively, *Oligohymenophorea* (OTU03 and 04) were the most abundant eukaryotic members in the sludge biomass in all reactors. Testate amoeba, including OTU06 and OTU07, were not detected beyond week 5, by which time compact aggregates had formed.

Non-metric multi-dimensional scaling (nMDS) visualization of the eukaryotic communities during granulation demonstrated a high level of dissimilarity between the flocs at week 0 and granules at week 1 (Fig. S1c). Based on sludge particle size, the determinant of granulation, the majority of the eukaryotic OTUs, except for *Salpingoecidae* (OTU10), were positively correlated with the floccular particle size (Fig. 3e). In contrast, both *Oligohymenophorea* (OTU01 and 03) demonstrated a strong positive correlation with the particle size during the initiation and maturation phase. The remaining eukaryotic OTUs had a negative correlation during both the initiation and maturation phases (Fig. 3e).

**Fig. 4:**
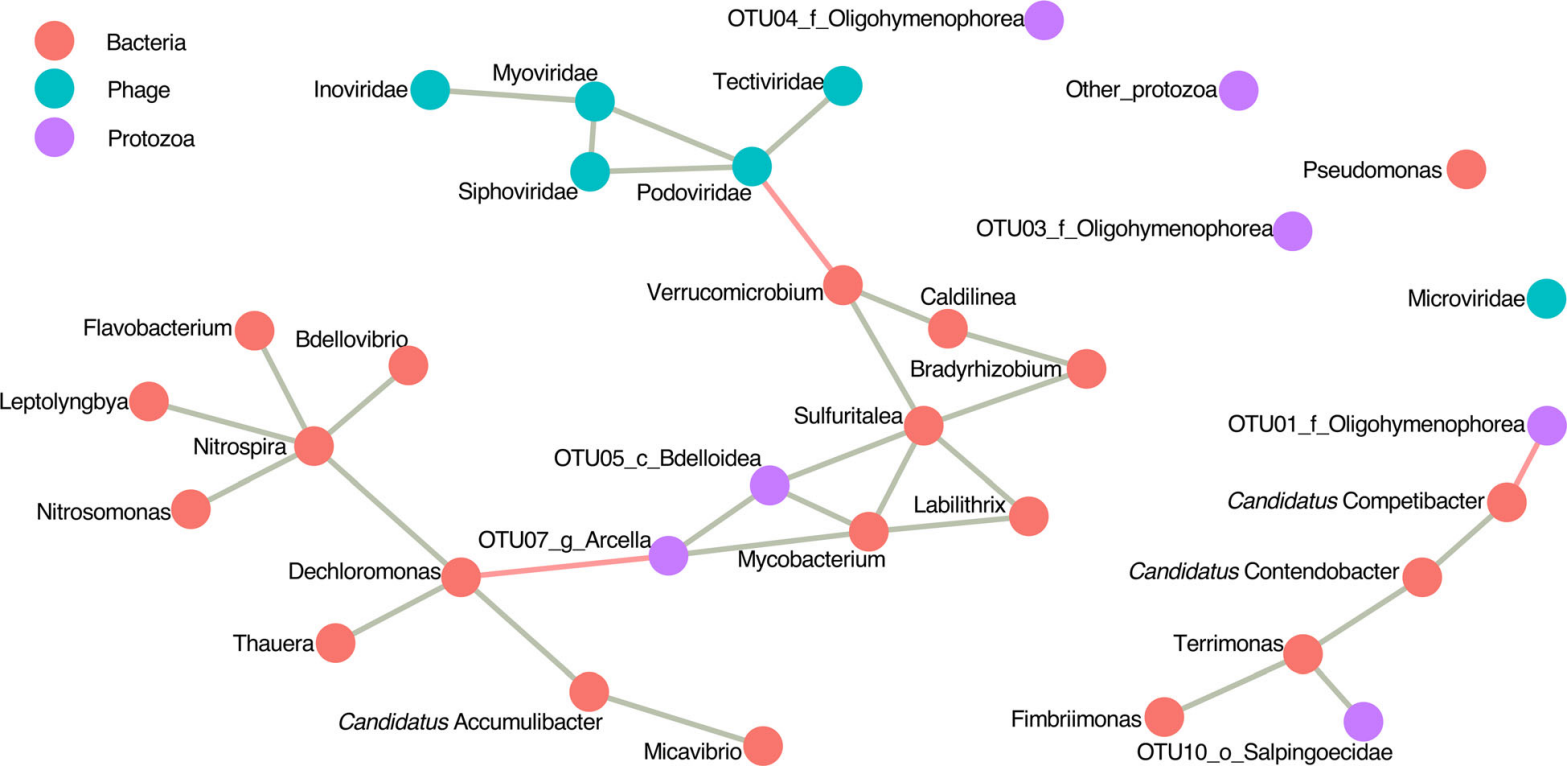
Microbiome network of the SBR showing potential interactions between the bacteria, protozoa and phage communities. The different groups are differentiated by colour. The lines connecting nodes (edges) are coloured depending on whether the correlation is positive (green) or negative (red)

A network analysis was undetaken to identify taxa that were possibly interacting with each other over the period of reactor operation (Fig. 4). There were 4 protozoan members that were correlated to the bacterial members of the reactor community. OTU01 and OTU07 were negatively correlated to ‘*Candidatus* Competibacter’ and *Dechloromonas* while OTU05 and OTU10 were positively correlated to *Sulfuritalea* and *Mycobacterium*, and *Terrimonas*, respectively. Most of the phages, except for Microviridae, were positively correlated to each other, with Podoviridae being the connecting node that is negatively correlated to *Verrucomicrobium*.

**Fig. 5:**
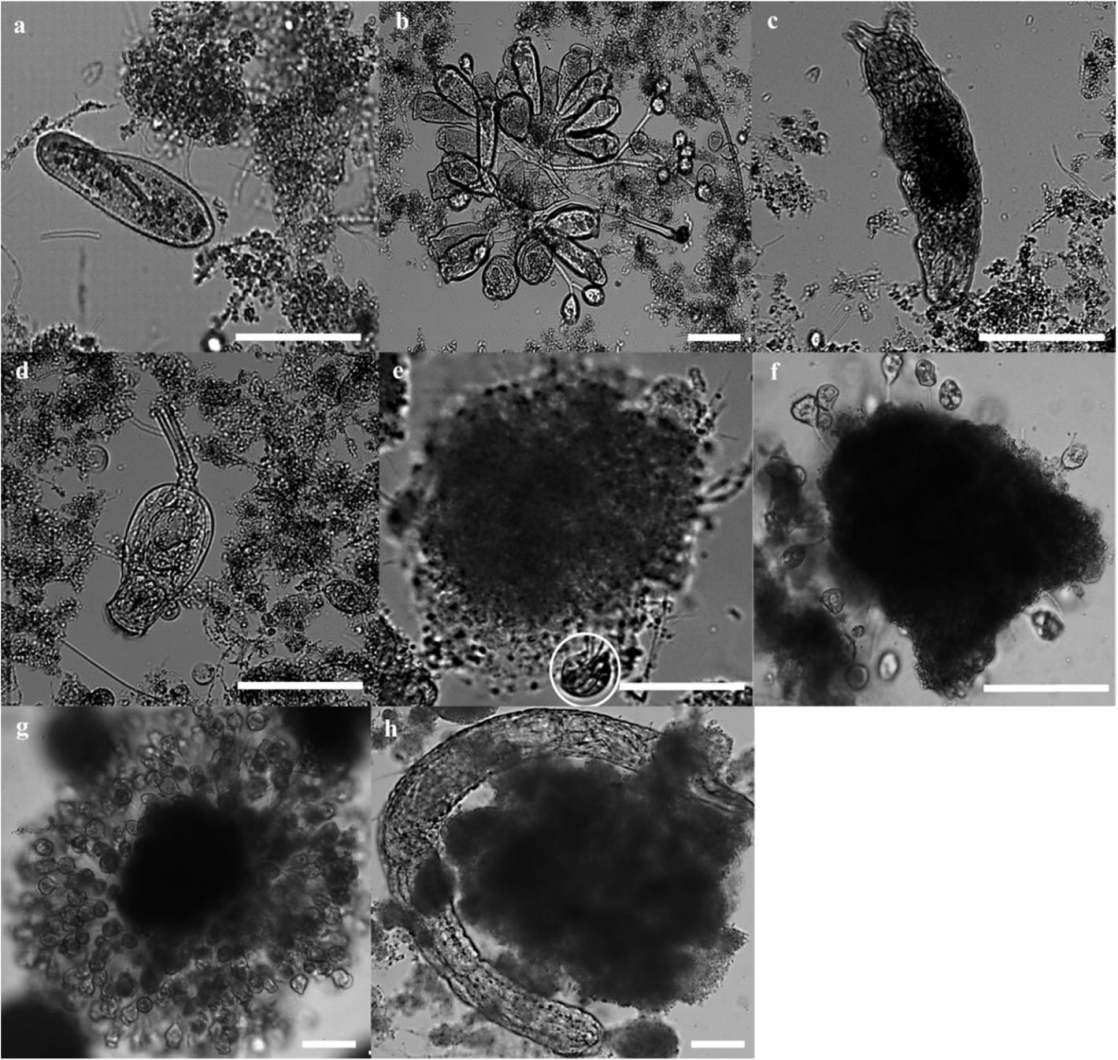
Micrographs of protozoa and metazoa in floccular and granular sludge. **a** A swimming ciliate, *Paramecium* spp*.* and **b** sessile ciliates*.* **c** Metazoa such as tardigardes and **d** large rotifers from the genus *Euchlanis.* **e** Crawling ciliates (circled in white) were commonly sighted. **f** and **g** Sessile ciliates attached on the surface of granules. (Bar, 50 μm)

While sequencing provided insights into the eukaryotic communities in the sludge during granulation, microscopic observations were also performed to determine the presence of protozoa and other eukaryotes. Microscopic observations of sludge have also been utilized in membrane bioreactors to compliment sequencing data observations [30]. Swimming ciliates that were most likely *Paramecium* spp*.* were observed within the floccular sludge (Fig. 5a), while sessile ciliates were attached to the surfaces of the flocs (Fig. 5b). These ciliates represent the *Oligohymenophorea* OTUs detected by sequencing (Fig. 3d). Metazoans such as tardigardes (Fig. 5c) and rotifers (Fig. 5d) were frequently observed in the floccular sludge with crawling ciliates such as *Aspidisca* sp., circling the Phase I flocs (Fig. 5e). These rotifers were likely to be represented by OTU05 and 08 as identified in the sequencing data (Fig. 3d). These observations clearly indicated that the inoculum floccular sludge had a diverse community of protozoa present prior to seeding into the SBRs. Upon the formation of compact aggregates at Phase II, no swimming ciliates or large eukaryotes were observed, although rotifers were still occasionally present. Upon granule formation at Phase III, the frequency of crawling ciliates decreased significantly, while sessile ciliates were frequently observed on the granule surfaces (Fig. 5f and g). The abundance of sessile ciliates, as determined by microscopy, were also reflected in the sequencing data where there were increases in *Oligohymenophorea* associated sequences (i.e OTU01, 03 and 04) in most reactors as granules formed.

### Development of aerobic granules from untreated and thiram treated floccular sludge

**Fig. 6:**
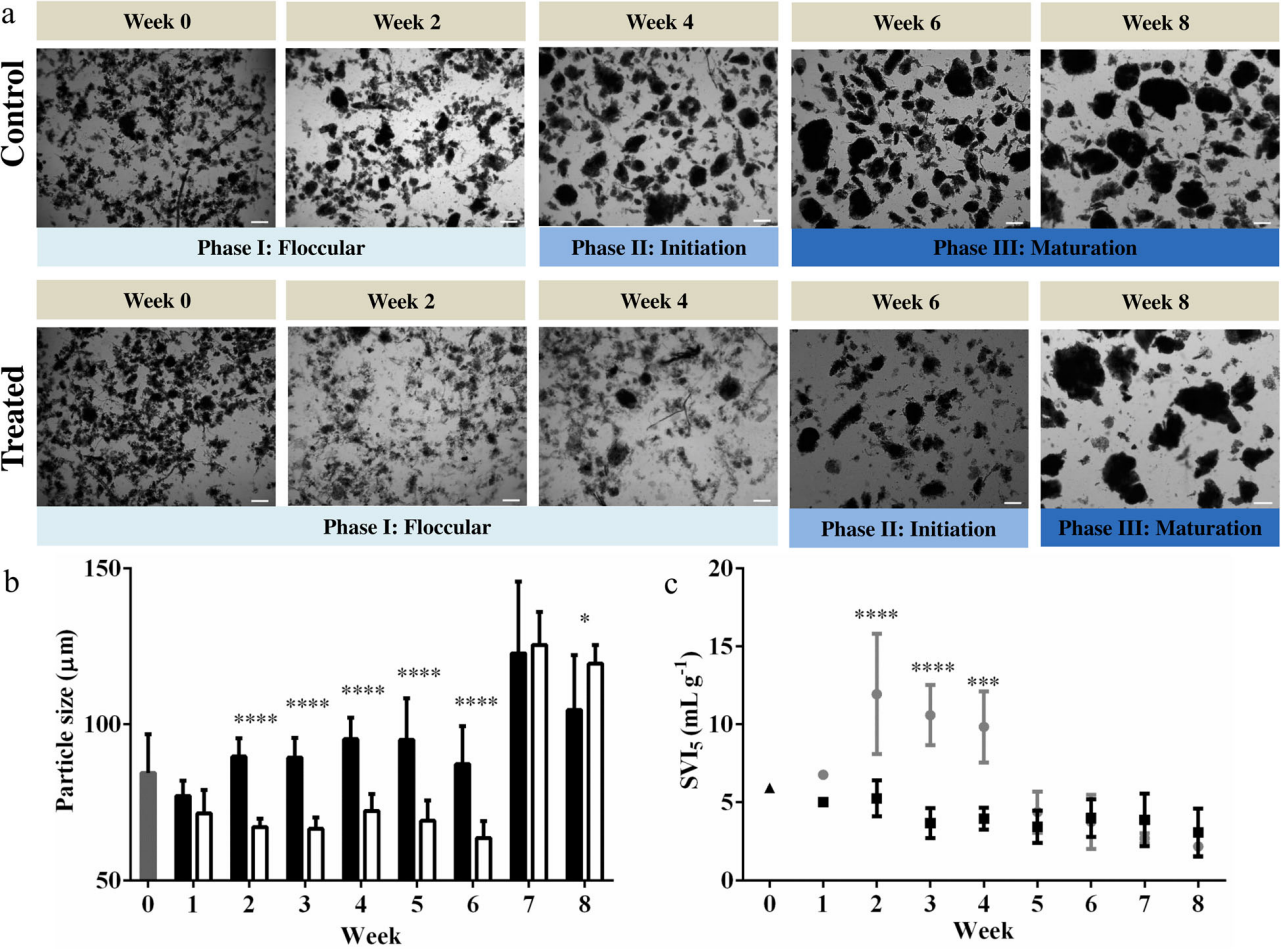
Development of granules from untreated and thiram treated floccular sludge. **a** Micrographs of control and thiram treated floccular sludge treated. **b** Mean particle size of seed (dark grey, week 0), control (black) and treated sludge (white) over 8 weeks. **c** SVI_5_ of seed (black triangle), control (black square) and treated (grey circle) sludge. Error bars represent standard deviation (*n* = 3) * and **** denote significant differences (One-way ANOVA: *P*-value ≤0.05 and 0.0001, respectively). Magnification × 40 (Bar, 200 μm)

Six mSBRs were seeded with activated floccular sludge and operated under conditions that were optimal for the aerobic granulation process over a period of 8 weeks. To investigate the role of protozoan predation in aerobic granulation, protozoa were removed from the floccular sludge by the addition of 20 mg L^− 1^ thiram to the mSBRs and DMSO was added as a control. The concentration of thiram was previously optimized to minimize any negative effects on the viability of bacteria in the floccular sludge (data not shown). Microscopic observations of control floccular sludge indicated that the conversion of floccular into granular sludge began at week 4 (Fig. 6a). Compact aggregates were observed in the initiation phase and these aggregates continued to expand in size. The sludge entered the maturation phase at week 6 and remained in this phase until the end of the experiment at week 8 (Fig. 6a). In contrast, thiram treated sludge did not initiate granulation until week 6 and only started to mature by week 8 (Fig. 6a).

As the volumes of the mSBRs were too low to allow for particle sizing by the particle size analyser, particle sizing was obtained by quantitative image analysis. The initial mean sludge particle sizes were 84.36 ± 12.41 μm (Fig. 6b) and by week 2, the control sludge mean particle size was 89.61 ± 5.94 μm, while the treated sludge was significantly smaller, 67.02 ± 2.65 μm, than the control sludge (Fig. 6b). By week 7, the treated sludge was 125.42 ± 10.60 μm, which was similar to the control sludge particles, 122.71 ± 23.00 μm (Fig. 6b). By week 8, there was a slight decrease in the control sludge (104.60 ± 17.57 μm), while the thiram treated sludge was significantly larger, 119.36 μm ± 6.05 μm (Fig. 6b).

The SVI_5_ of the treated sludge was significantly higher than the control sludge from weeks 2 to 4 (Fig. 6c), suggesting that the thiram treated sludge was less dense and compact and hence required a longer settling time compared to the control sludge. However, from week 5 onwards, the SVI_5_ for the thiram treated sludge decreased and was not significantly different from the control sludge.

### Effects of thiram treatment on microbial communities during aerobic granulation

**Fig. 7:**
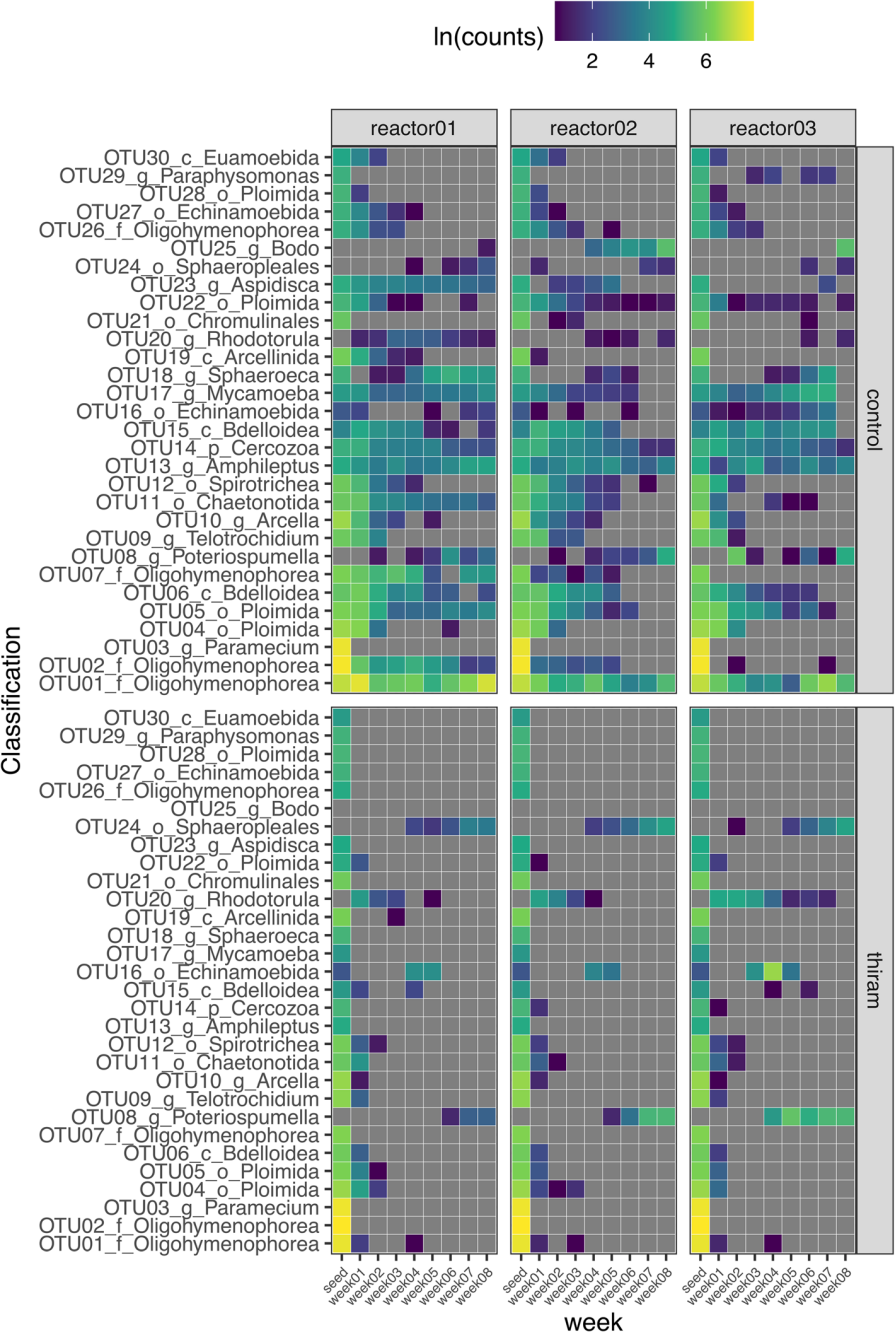
Abundance of eukaryotic populations in control and treated mini-SBRs over 8 weeks of granulation. There were 3 control and 3 thiram treated mini-SBRs. The number of sequences per OTU in both control and treated were natural log transformed. These 30 OTUs represented approximately 92% of the total eukaryotic sequences

The microbial communities in the two sludge types were compared by metacommunity sequencing of the V5 region of the 16S and 18S rRNA genes using the Ribotagger method [31]. A total of 30 OTUs, representing approximately 92% of the eukaryotic communities were selected for analysis. Within the inoculum sludge, the eukaryotic communities were dominated mainly by ciliated protozoa OTUs, e.g. OTUs 01, 02 and 03 (Fig. 7). As granulation progressed in the control mSBRs, the abundances of these OTUs were consistent, with *Oligohymenophorea* (OTU01) being the most dominant. Both *Oligohymenophorea* OTUs 02 and 07 showed a gradual decline in abundance while *Oligohymenophorea* (OTU26) was not detected beyond week 5. Swimming ciliates from the genus *Paramecium* (OTU 03) were not detected after week 1 (Fig. 7). In contrast to the swimming ciliates, crawling ciliates from the genus *Aspidisca* (OTU 23) were relatively abundant during granulation. However, these protozoan OTUs were mostly not detected after week 1 in the thiram treated sludge (Fig. 7). Interestingly, two flagellate associated OTUs, OTU08 and 24, increased in abundance in the treated sludge from week 4 onwards.

Metazoan OTUs representing rotifers, e.g. OTUs 04, 05, 06, 15 and 22, were also detected in relatively high abundance in the control sludge and were present throughout the entire granulation process (Fig. 7). In contrast, these rotifers were only detected at low abundance during the first 3 weeks in the treated sludge and were mostly not detected beyond week 4. Eukaryotic communities in the control sludge did not changed drastically over time (Fig. S3a). However, eukaryotic communities in the thiram treated sludge diverged over time and were distinctly different from the control sludge from week 1 to 5 (Fig. S3a). This was likely due to the absence several dominant protozoan OTUs including OTU01, 02 and 07. Interestingly, the eukaryotic communities in the control and treated sludge began to converge from week 6 onwards, which was likely due to the resurgence of protozoan OTU08 and OTU24 (Fig. S3a).

Bacterial OTUs were also analyzed to determine if the absence of predators had any impact on the bacterial communities during aerobic granulation. Based on nMDS visualization, two distinct clusters were observed which indicated dissimilarities between the control and thiram treated bacterial communities during granulation (Fig. S3b). The bacterial communities remained relatively similar from week 2 to week 8 in the control sludge while the bacterial communities in the thiram treated sludge continue to change on a weekly basis (Fig. S3b). In addition, there was no significant difference in the microbial communities between replicates of the control or thiram sludge due to close clustering in each week (Fig. S3b).

**Fig. 8:**
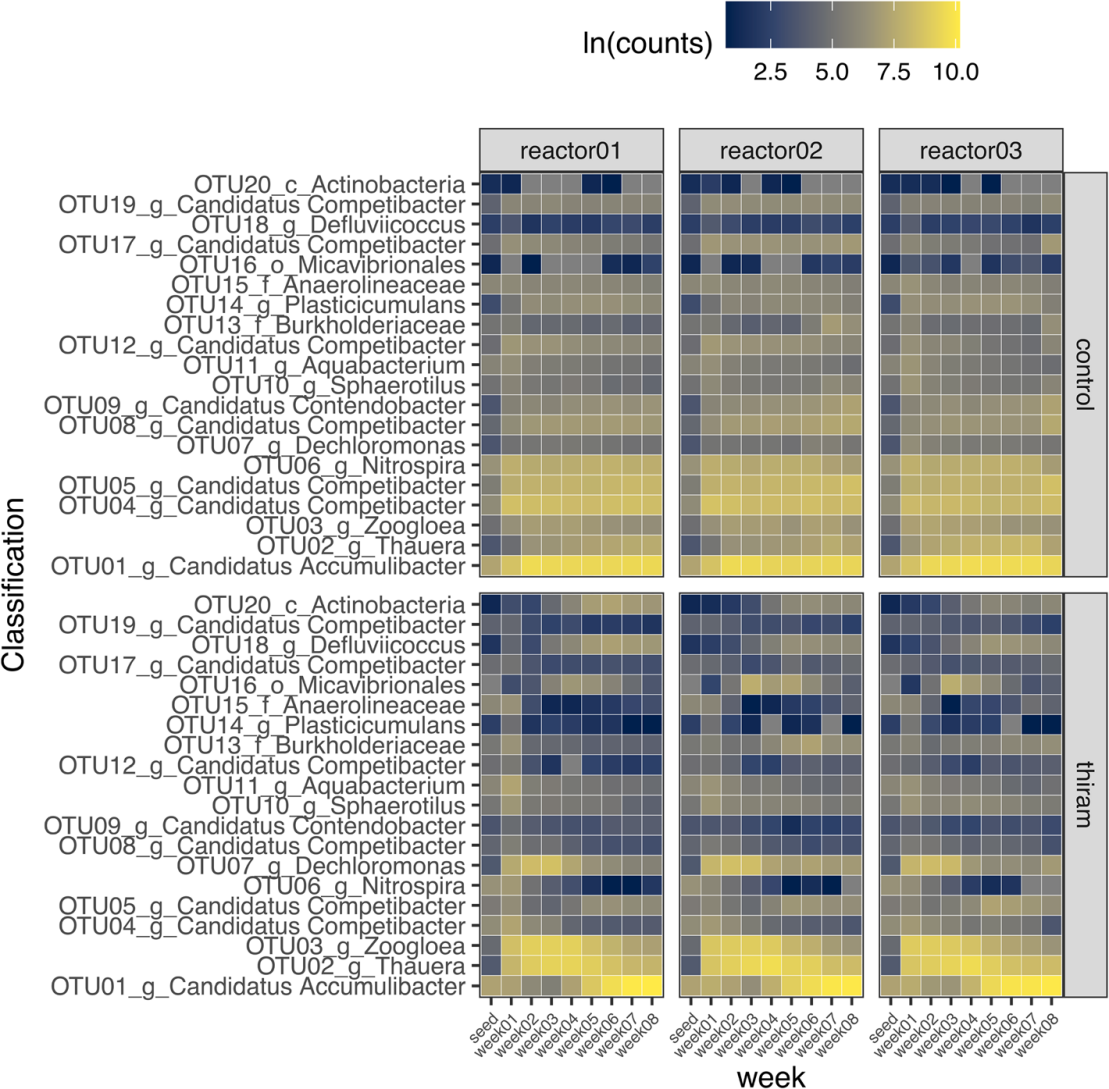
Abundance of top 20 abundant bacterial OTUs in control and treated mini-SBRs over 8 weeks of granulation. There were 3 control and 3 thiram treated mini-SBRs. The number of sequences per OTU in both control and treated were natural log transformed before analysis

In the control sludge, the bacterial communities were dominated mainly by PAOs such as ‘*Candidatus* Accumulibacter’ (OTU01), GAOs such as ‘*Candidatus* Competibacter’ (OTU04 and 05) and *Nitrospira* (OTU06) throughout 8 weeks of aerobic granulation (Fig. 8). The abundance of other bacterial members such as *Zoogloea* (OTU03)*, Thauera* (OTU02), *Dechloromonas* (OTU07), ‘*Candidatus* Competibacter’ (OTU12, 17 and 19), ‘*Candidatus* Contendobacter’ (OTU09), *Defluviicoccus* (OTU18) and *Actinobacteria* (OTU20) remained relatively consistent during granulation (Fig. 8). In contrast, there was a decrease in the abundance of ‘*Candidatus* Accumulibacter’ (OTU01) and ‘*Candidatus* Competibacter’ (OTU04 and 05) from week 1 in the thiram treated sludge. The genus *Nitrospira* (OTU06) also demonstrated decline in abundance from week 1 onwards with no sign of recovery (Fig. 8). The decrease in abundance was also observed in ‘*Candidatus* Competibacter’ (OTU12, 17 and 19) and ‘*Candidatus* Contendobacter’ (OTU09) (Fig. 8). Interestingly, there were several bacterial OTUs such as *Zoogloea (*OTU03)*, Thauera* (OTU02), *Dechloromonas* (OTU07) and *Defluviicoccus* (OTU18) that increased in abundance from week 1. However, as ‘*Candidatus* Accumulibacter’ (OTU01) began to gradually increase in abundance from week 5, the abundance of *Zoogloea (*OTU03)*, Thauera* (OTU02), *Dechloromonas* (OTU07) decreased. In contrast, OTU18 and 20 continued to gradually increase in abundance from week 5 onwards. ‘*Candidatus* Accumulibacter’ (OTU01) increased in abundance in the thiram treated sludge as it entered Phase II of granulation, where compact aggregates were formed.

## Discussion

The abundance of ‘*Candidatus* Accumulibacter’ increased progressively during the granulation process together with ‘*Candidatus* Competibacter’, ‘*Candidatus* Contendobacter’ and *Nitrospira* which were present at relatively high abundance (Fig. 2). Their higher abundance during granulation over other bacterial members suggests their close association with denser flocs or granules with better settling characteristics. This is similar to other biological nutrient removal systems where ‘*Candidatus* Accumulibacter’ and ‘*Candidatus* Competibacter’ were among the most abundant in the granular sludge community [25, 32, 33]. The higher abundance of ‘*Candidatus* Accumulibacter’ compared to ‘*Candidatus* Competibacter’ observed during the maturation phase could be partly due to the presence of propionate in the synthetic feed, which ‘*Candidatus* Accumulibacter’ has been shown to utilize/uptake more efficiently than ‘*Candidatus* Competibacter’ [34].

As ‘*Candidatus* Accumulibacter’ is often enriched for their capacity to remove phosphorus, their high abundance can increase their susceptibility to phage attack. It has been reported that abundant bacteriophage-like particles coupled with declining abundance of ‘*Candidatus* Accumulibacter’ and lysed ‘*Candidatus* Accumulibacter’ cells strongly suggested that phage infection was the main cause for ‘*Candidatus* Accumulibacter’ mortality [18]. Furthermore, the addition of bacteriophage-containing supernatant to other wastewater sludge also demonstrated similar decline in the abundance of ‘*Candidatus* Accumulibacter’, resulting in poor phosphorus removal [18]. These observations strongly suggest that phage infection can alter bacterial communities and their functionality of the systems in which they are present. The presence of certain bacteriophage families may be an indication that phage predation and the lysis of specific bacteria are required during the process of granulation. The change in abundance of Microviridae and Podoviridae (which are generally lytic phages) and Siphoviridae (temperate phages) were positively correlated with the increase in sludge particle size (Fig. 3b) and Microviridae were associated with changes in bacterial composition (Fig. 3c). While network analysis suggested that Podoviridae may be selectively predating on *Verrucomicrobium* (Fig. 4), the bacterial targets in flocs and granules of Microviridae, Podoviridae and Siphoviridae remain unclear. *Verrucomicrobia* are common in many wastewater treatment plants though a specific function has not been attributed to them [35, 36].

Although the role of lytic phages in the formation of aerobic granules is still unknown, a recent study demonstrated that bacteriophage-mediated extracellular DNA release was found to be vital for the structural stability of smaller aerobic granules [20]. Extracellular DNA containing clustered regularly interspaced short palindromic repeats (CRISPR) spacers was found to be part of the aerobic granular structure and extensive flocculation happened upon treatment with DNase I [20]. In our study here, the sequences associated with lytic phages from Microviridae and Podoviridae were constantly detected during the granulation process, suggesting that bacterial hosts associated with these families of lytic phages were present and phage lysis were actively ongoing throughout granulation. The increased abundance of Inoviridae (which are generally non-lytic filamentous bacteriophages) [37] in the reactor effluent at weeks 5, 7, 8 and 9 (Fig. 3a) coincided with the appearance of compact aggregates (Fig. 1). Inoviridae also seemed to influence changes in the bacterial community (Fig. 3c), in particular for weeks 4 to 8, when the sludge was in the initiation to early maturation stages of granulation. Aerobic granule development has been suggested to be similar to biofilms that are surface associated [38], both containing EPS in their structure [26] as well as adhesion and cell-cell contact [39]. As for surface-associated biofilms, it is possible that filamentous phages play a role as a structural component in the granule as well. An example would be for the Pf4 filamentous phage in *Pseudomonas aeruginosa* biofilms where the phage organises the biofilm matrix into a liquid crystal structure that has increased viscosity and adhesion [27].

Other than bacteriophages, both microscopy and sequencing data analysis demonstrated an abundance of protozoa present during the floccular phase (Fig. 3d and 4). Previous studies also reported a high abundance and diversity of protozoa in floccular sludge [40–42]. While protozoan predation in activated sludge has been suggested to facilitate increased biofilm production, a negative or lack of correlation was observed between the ciliated protozoa and particle sizes during the floccular phase. In contrast, there was a positive correlation between the flagellate family *Salpingoecidae* and the floccular particle size (Fig. 3e). Flagellates commonly predominate the activated sludge in the early stages as they consume lesser energy required for growth compared other larger protozoa such as ciliates. Interestingly, this family of flagellates was continuously detected throughout granulation and did not demonstrate any positive correlation with the particle sizes in the initiation or maturation phases. However, as compact aggregates formed and expanded during the initiation phase, sequences associated with *Oligohymenophorea* continued to be detected at relatively high abundance. While these sequences cannot clearly define the types of ciliated protozoa, microscopy analysis indicated that crawling and sessile ciliates increased in abundance. Grazing by crawling ciliates has been reported to stimulate the growth of microcolonies for surface attached biofilms [43]. In addition, activated sludge flocs that were co-cultured with crawling ciliates from the genus *Aspidisca* demonstrated an increase in floc particle size and compactness [44]. Moreover, the motility of crawling ciliates such as *Chilodonella* can dislodge cells from biofilms [43, 45]. These dislodged cells could then become a food source for the filter-feeding sessile ciliates. As more compact and dense granules formed, crawling ciliates are likely outcompeted by sessile ciliates whose growth is favored by an increase in granule surface area.

Sequencing analysis demonstrated ciliates from the class *Oligohymenophorea* were present throughout the granulation process (Fig. 3d). The class *Oligohymenophorea* consist of several subclasses such as *Peritrichia* that represent a distinctive group of sessile ciliates which are hypothesised to play an important role in granulation [22, 24, 46]. Sessile ciliates were often observed on the surfaces of activated sludge flocs and aerobic granules [21, 24, 47]. While sessile ciliates were observed microscopically on granules (Fig. 5f and g), there were no sequences from sessile ciliates that were classified to the genus level. Nonetheless, positive correlation with particle size during the initiation phase for the OTU 01 and 03 representing *Oligohymenophorea* suggests that these two classes of ciliated protozoa could play a more important role during the initiation phase of aerobic granulation rather than in the formation of mature granules. It is also possible that the change from flocs to granules acted as a form of selection pressure on the protozoan community, which also led to a significant reduction in the abundance and diversity of protozoa. For example, the presence of abundant, free-swimming protozoa will exert predation pressure on free swimming bacteria, which is known to promote biofilm formation such as compact aggregates and aerobic granules. However, the formation of biofilms simultaneously increases availability of substratum for colonization of sessile ciliates and surface grazing by crawling ciliates while reducing availability of free swimming bacteria for predatory flagellates and free swimming ciliates. Overall, the data suggest that the formation of aggregates favoured the growth of crawling and sessile ciliates while the compact and large granules favoured the colonisation of sessile ciliates.

Weber et al. [24] hypothesised that sessile ciliates could also act as nucleating agents for the attachment of bacteria. To investigate the role of protozoan predation in granulation, thiram was added into floccular sludge in this project. The resulting eukaryotic community in the thiram treated sludge was significantly different from the non-treated sludge where OTUs corresponding to protozoa were rarely detected by week 2 in the thiram treated sludge (Fig. 7). Importantly, there was very low or no detection of ciliated protozoa in the treated sludge. Without these ciliated protozoa particularly sessile ciliates, bacteria attachment was affected as the significant reduction of sessile agents contributed towards a loss of nucleating agents. It is likely that the absence of protozoa was responsible for the delayed increase in particle size. Protozoa have also been demonstrated to excrete growth stimulating products which could potentially induce flocculation [48]. For example, the co-incubation of activated sludge bacteria with sludge protozoa composed of attached, crawling ciliates, flagellates and amoeba for 48 h, resulted in biofilms that had 2000% more biomass than the biofilms that were not exposed to protozoan predation [11]. In addition, polymeric substances such as extrusomes, cellular debris and undigested residues secreted from protozoa could also facilitate aggregation between bacterial flocs [49]. Hence, the absence of protozoan predation could have reduced the selection pressure aggregation, resulting in less dense flocs that settle poorly. Poor settling of the treated sludge could also have resulted in larger losses of sludge biomass during discharge. This finding corresponded to previous observations where ciliated protozoa were important for the formation of compact aggregates which leads to granule formation.

In the absence of protozoan predators, the floccular sludge demonstrated poor compactness and settling. Both ‘*Candidatus* Accumulibacter’ and ‘*Candidatus* Competibacter’ were replaced by the genera *Thauera* and *Zoogloea* as the dominant members of the bacterial community in the treated floccular sludge in Phase I. The ‘*Candidatus* Accumulibacter’ could have also been replaced by both genera of *Thauera* and *Dechloromonas*, which are PAOs that are also capable of denitrification [50]. The proliferation of *Zoogloea* was likely due to insufficient retention of sludge [32, 51]. *Zoogloea* are floc-forming bacteria that produce aggregates enveloped in gelatinous matrixes that could have initiated the formation of compact aggregates. However, the initiation phase was delayed in the thiram treated reactors despite the abundance of Zoogloea associated sequences and it is possible that the delay in initiation was due to the absence of protozoa.

While there was a delay in initiation, the thiram treated sludge ultimately granulated by week 8 and we hypothesised that it could be largely attributed to the emergence of ‘*Candidatus* Accumulibacter’ after week 5. In contrast, the control sludge, which was dominated mainly by ‘*Candidatus* Accumulibacter’ and ‘*Candidatus* Competibacter’, entered the initiation and granulation phase earlier at weeks 4 and 6, respectively. These observations further support that the high abundance of ‘*Candidatus* Accumulibacter’ and ‘*Candidatus* Competibacter’ are strong potential contributors towards the formation of aerobic granules. Other than bacteria, it is also likely that protozoan predation plays a partial role in enhancing the formation of aerobic granules by reducing the time to initiate compact aggregates formation.

The limitations of this study are that while the mechanistic link between protozoan predation and granulation was directly tested, the effect of bacteriophages on granulation is correlative. In addition, the current sequencing data is unable to define *Oligohymenophorea* sequences to the genus or species level. Hence, any true impact on granulation from these ciliates will require isolation of specific protozoa and subsequently adding them back in significant amounts in floccular or granular sludge. Future work should address these issues by isolating phages and protozoa from key points in the granular development (e.g. initiation phase) and adding back these phages to either floccular or granular sludge to see their more direct impacts on granulation.

## Conclusions

Predation by bacteriophages and protozoa can influence the diversity and structure of the bacterial community. The alteration of bacterial community composition subsequently affected the rate of granulation of floccular sludge. While physical parameters such as settling time have significant effects on promoting granulation, we have also demonstrated the potential role of bacteriophage and protozoa in promoting granulation through physical means such as bacterial attachment on phage filaments or sessile ciliates.

## Methods

### Sequencing batch reactor setup and operation

To characterize the protozoan communities during aerobic granulation, four independent SBRs were seeded with activated floccular sludge from the Ulu Pandan Wastewater Treatment Plant, Singapore, as previously described [52, 53]. Briefly, each SBR had a final working volume of 2 L and was operated in a 6 h cycle comprising two different phases: Phase I - feeding (8 min), anaerobic (60 min), aerobic (80 min at day 0, with a gradual increase to 95 min by week 5) and anoxic (40 min at day 0, with a gradual increase to 50 min by week 5); Phase II - feeding (2 min), anaerobic (30 min), aerobic (40 min at day 0 and gradual increase to 70 min by week 5) and anoxic (30 min). Each cycle was completed with a settling stage (120 min at day 0, with a gradual decrease to 5 min by the end of week 6) and a 10 min decanting stage. The settling time was maintained at 5 min per cycle from week 6 onwards.

A total volume of 1 L of synthetic wastewater was supplied to each SBR by Phase II and 1 L of effluent was discharged at the completion of each cycle. Synthetic wastewater was prepared as previously described [54, 55]. Dissolved oxygen (DO) levels were maintained at 0.0 mg L^− 1^ during anaerobic phases via intermittent nitrogen sparging and maintained between 3.0 to 4.0 mg L^− 1^ during aerobic phases by compressed air sparging. Sparging of both nitrogen and air provided complete mixing of the sludge and the hydrodynamic shear force required for aerobic granulation. The pH of each SBR was maintained between 6.8 and 8.2 by dosing with 0.1 M HCl and 0.1 M NaOH as required. Both pH and DO levels were monitored by inline probes connected to a programmable logic controller (PLC).

Mixed liquor suspended solids (MLSS) and mixed liquor volatile suspended solids (MLVSS), were determined using APHA standard engineering methods [56]. Sludge density and compactness was measured by sludge volumetric index at 5 min (SVI_5_) as described [7]. Sludge particle sizes were determined using a laser diffraction particle size analyser (SALD-3101, Shimadzu, Japan) and their morphology was recorded by light microscopy (Primo Star, Carl Zeiss, Germany). At the end of each cycle study, well-mixed sludge samples of 1 mL were collected from each reactor at the end of Phase II anoxic stage. These sludge samples were centrifuged at 8000 *g* for 5 min and snap frozen in liquid nitrogen prior to storage at − 80 °C.

### Total genomic DNA extraction from aerobic granular sludge

One milliliter of suspended sludge was pelleted by centrifugation for 10 min at 10,000 *g*. The total genomic DNA was extracted using the sludge pellet with the FastDNA™ SPIN Kit for Soil (MP Biomedical, USA) mostly according to the manufacturer’s guidelines. Homogenization was performed twice in the FastPrep® Instrument for 40 s at a speed setting of 6.0. The extracted genomic DNA was then cleaned up using the Genomic DNA Clean & Concentrator (Zymo Research, USA) according to the manufacturer’s guidelines. The concentration of the DNA was quantified using the dsDNA HS Assay Kit and the Qubit® 2.0 Fluorometer (Life Technologies, USA) before sequencing on the Illumina HiSeq as 250 bp paired end reads.

### Total metagenome analysis of the aerobic granular sludge

The quality of the metagenomic reads was assessed using FastQC (v 0.11.5) before it was adapter and quality trimmed using BBMap (v 36.38) [57]. Contigs were co-assembled using MEGAHIT (v 1.0.6–3) [58] with the meta-sensitive preset mode before ORF prediction was done using the meta mode of Prodigal (v 2.6.3) [59]. Using nucleic acid ORF sequences, redundancy was removed using cd-hit-est (v 4.6.8) [60] with the options for 95% sequence identity and word length of 10. The non-redundant ORF sequences were then used in a protein homology search using the Blastx function of DIAMOND (v 0.8.22) [61] against the NCBI nr database. Based on the Blastx output, the lowest common ancestor (LCA) annotation for the contigs were performed using MEGAN6 Community Edition (v 6.8.12) [62]. To obtain the contig abundance table, the metagenome reads were mapped to the co-assembled contigs using Bowtie2 (v 2.2.6) [63] before read coverage was obtained with the idxstats function of Samtools (v 1.3.1) [64]. The contig abundance and LCA-annotated contigs were then analysed using Phyloseq (v 1.22.3) [65] in R.

### Viral fraction sampling and concentration

During SBR operation, the viral fraction was collected and concentrated as previously described [52]. Briefly, effluent from each reactor was discharged into their respective containers before transferring into 25 L carboys. Samples were collected from weeks 1 to 10 of the study. The initial filtrate was obtained by passing through a 25 μm filter bag (Puridea, Singapore) to remove any suspended biomass before storing at 4 °C. The filtrate was pooled over 4 d to obtain 20 L, at which time, 2 mL of DNase I (200 U/mL) (Calbiochem, USA) was added to the samples to digest any extracellular DNA. Bacteria were then removed by passing the filtered effluent through a 0.2 μm Sartocon Slice Disposable tangential flow filter (TFF) (Sartorius Stedim, Germany). To concentrate the viral fraction, the permeate was concentrated using a 100 kDa Sartocon Slice Dispostable TFF (Sartorius Stedim, Germany). In this process, the fluid phase and particles smaller than 100 kDa were removed while the viral fraction remained in the reservoir. To elute the viral fraction, SM buffer (100 mM NaCl, 8 mM MgSO_4_, 50 mM Tris-Cl at pH 7.5) was added to the phage reservoir until the volume was reduced to a 100 mL. The viral fraction was further concentrated using the Vivaspin 20 100,000 MWCO Centrifugal Concentrators (Sartorius Stedium, Germany) by centrifuging at 5000 *g* for 30 min at 4 °C until a volume between 2 to 3 mL was obtained and stored at -80 °C as 200 μL aliquots.

### Bacteriophage nucleic acid extraction and multiple displacement amplification

Nucleic acids were extracted using the QIAamp MinElute Virus Spin kit (Qiagen, Germany) from 200 μL of concentrated viral fraction according to manufacturer’s guidelines. The viral DNA was used for whole genome multiple displacement amplification (MDA) using random hexamers with the illustra GenomiPhi V2 DNA amplification kit (Cytiva, USA) according to the manufacturer’s guidelines. The amplified DNA was then purified using the ethanol precipitation method [66]. Briefly, sodium acetate was added and mixed to the amplified sample to a final concentration of 0.3 M at pH 5.2. Two volumes of cold 100% molecular grade ethanol was added and incubated overnight at − 20 °C. After incubation, the sample was centrifuged at 15,000 *g* for 30 min and the supernatant was removed. One mL of 70% ethanol was then added and incubated at − 20 °C for 2 h before centrifuging at 15,000 *g* for 30 min to pellet DNA. The supernatant was discarded and the pellet air dried for 5 min before resuspension in sterile dH_2_O.

### Analysis of the viral fraction

The quality of the metavirome reads was assessed using FastQC (v 0.11.5) before adapter and quality trimming using BBMap (v 36.38) [57]. Contig co-assembly was done using the MEGAHIT (v 1.0.6–3) meta-sensitive preset mode [58] before doing ORF prediction using the Prodigal (v 2.6.3) meta mode [59]. The viral ORF sequences were used in a protein homology search using the DIAMOND (v 0.8.22) Blastp program against the A CLAssification of Mobile genetic Elements (ACLAME) database. Based on the output, the LCA annotation for the viral contigs were obtained using MEGAN6 Community Edition (v 6.8.12) [62]. The metavirome reads were then mapped to the viral contigs using Bowtie2 (v 2.2.6) [63] before obtaining read coverage using the idxstats function of Samtools (v 1.3.1) [64]. The LCA annotated viral contigs and their abundances were then used for downstream analyses.

### Mini-sequencing batch reactors setup and operation

Mini-SBRs (mSBR) were seeded with activated floccular sludge from the Ulu Pandan Wastewater Treatment Plant, Singapore. For floccular sludge experiments, both controls and treatments were performed in triplicate while granular sludge experiments were performed in duplicate. Each mSBR had a final working volume of 30 mL and was operated in a 6 h cycle: feeding (10 min), anaerobic (100 min), aerobic (110 min at day 0, with a gradual increase to 120 min by the end of week 1) and anoxic (100 min) phases. Each cycle was completed with a settling stage (30 min at day 0, with a gradual decrease to 20 min by the end of week 1) and a 10 min decanting stage. The settling time was maintained at 20 min per cycle from the end of week 1 onwards.

Synthetic wastewater (15 mL) was fed to each mSBR in Phase II and 15 mL of treated effluent was discharged at the end of the cycle. For the inhibition of eukaryotes, thiram (Sigma Aldrich, Germany) was dissolved in dimethyl sulfoxide (DMSO) to obtain a stock solution of 20 g L^− 1^ for treatment of the floccular sludge. Thiram has been shown to inhibit protozoa with minimal impacts on bacterial activities [67]. Based on optimization studies, thiram was added to each reactor once per day after feeding to obtain a final concentration of 20 mg L^− 1^ (data not shown), while DMSO was added to control mSBRs. Both DMSO and thiram treatment of sludge was completed by week 2. Both control and treated mSBRs were operated from weeks 3 to 8 without the addition of DMSO or thiram. To achieve anaerobic and aerobic conditions, nitrogen and compressed air were sparged intermittently into the mSBRs.

The average particle diameter of the floccular sludge was determined by analyzing images of sludge, taken in triplicate for each mSBR, on a weekly basis using ImageJ (National Institute of Health, USA). For enumeration of protozoa, triplicate 10 μL aliquots were removed from each mSBR and the numbers of protozoa determined using light microscopy (Primo Star, Carl Zeiss, Germany). Samples (1 mL) were collected from each mSBR at the end of Phase II anoxic stage, centrifuged at 8000 *g* for 5 min and snap frozen in liquid nitrogen prior to storage at -80 °C.

### RNA extractions for total RNA sequencing and analysis

Total RNA was extracted from sludge samples using the Soil, Fecal and Plant RNA kit (Zymo Research, USA) as described [68, 69], according to the manufacturer’s guidelines. Extracted RNA underwent a single round of DNase treatment to remove residual DNA (TURBO™ DNase kit; Invitrogen, Singapore). The quality of the extracted RNA was measured by spectrophotometry (Nanodrop; Thermo Scientific, USA). The concentration of RNA and residual DNA was determined by fluorometry (Qubit® 2.0 Fluorometer; Invitrogen, USA), using the Qubit® RNA broad range assay kit (Invitrogen, USA) and Qubit® DNA high sensitivity range assay kit respectively, following the manufacturer’s guidelines. In addition, the integrity of the RNA was determined using the RNA Analysis ScreenTape and 2200 Tapestation instrument (Agilent Technologies, Singapore) and reported as the RNA Integrity Number (RIN). These RNA samples were subsequently sent for RNA library preparation prior to pooling and sequencing on an Illumina HiSeq 2500 System (Illumina Inc.) using 100 bp paired-end (PE) sequencing as per the manufacturer’s guidelines.

### Total RNA sequencing and analysis

The microbial composition of the floccular and granular sludge was determined by analysis of the sequence data using the Ribotagger fast tag-based approach [31]. Briefly, universal recognition profiles that target bacteria, *Archaea* and eukaryotes were selected for each of the hypervariable regions of both 16S and 18S rRNA (e.g. V4, V5, V6 and V7) (Xie et al. [31]). These universal recognition profiles were used to scan the sequencing reads to obtain 33 nucleotides (nt) downstream of the primers (Xie et al. [31]). Each of these 33 nt tags were defined as a ribotag and each ribotag was screened against the SILVA database to map it to a known organism. Hence, each ribotag was used as a signature sequence to represent one operational taxonomic unit (OTU). Here, only the sequencing reads from the V5 regions of 18S rRNA were used to represent the abundance of protozoan communities. Based on the lowest number of total sequencing reads within the samples set, these V5 sequencing reads were randomly subsampled based on a seed value of 100 using the seqtk FASTQ program (https://github.com/lh3/seqtk).

### Network analysis

The combined abundance table of the bacterial, protozoan and viral communities were combined before it was loaded as a phyloseq object using Phyloseq (v 1.22.3) [65] in R. The network analysis was performed using the SpiecEasi package (v 1.1.0) [70]. Briefly, the abundance table was normalised using centered log-ratio transformation before inverse covariance estimation was done. The stability of the network was inferred using the package’s Stability Approach to Regularization Selection (StARS) criterion. The following parameters were used: method = “mb”, lambda.min.ratio = 0.05, nlambda = 100.

### Statistical analysis

Correlation studies for protozoa and bacteriophages were performed by calculating Spearman correlation coefficient using Prism (Graphpad 6.0). The resulting protozoa matrices were clustered hierarchically based firstly by obtaining the Bray- Curtis dissimilarity matrix and then clustering using the hclust function in vegan (v.5–6) [71] in R. The distance matrices were used for non-metric multi-dimensional scaling (NMDS) to determine the level of similarity or dissimilarity between of samples based no bacteria and eukaryotic communities.

## Supplementary Information

**Additional file 1.**

## Data Availability

The datasets generated and/or analysed during the current study are available in the DR-NTU (Data) repository, 10.21979/N9/TBOI0Y.
