# Supplementary material for: Microbial predation accelerates granulation and modulates microbial community composition
Source: BMC Microbiol. 2021 Mar 27;21:91. doi: 10.1186/s12866-021-02156-8 (PMC8004422; doi:10.1186/s12866-021-02156-8)
Supplement: Supplementary file 1 — Additional file 1. [file 12866_2021_2156_MOESM1_ESM.docx]

**Microbial predation accelerates granulation and modulates microbial community composition**

Siew Herng Chan*,^a,b^ Muhammad Hafiz Ismail*,^a,d^ Chuan Hao Tan,^a,c^ Scott A. Rice,^a,d,e#^ and Diane McDougald^a,e#^

^a^ Singapore Centre for Environmental Life Sciences Engineering, Nanyang Technological University, Singapore

^b^ Interdisciplinary Graduate School, Nanyang Technological University, Singapore

^c^ School of Materials Science and Engineering, Nanyang Technological University, Singapore

^d^ School of Biological Sciences, Nanyang Technological University, Singapore

^e^ The iThree Institute, University of Technology Sydney, Sydney, Australia

* Both authors contributed equally to this work

Running Head: Acceleration of granulation by microbial predation

#Corresponding author. Mailing address: The iThree Institute, University of Technology Sydney, Sydney, Australia, Building 4, Cnr Thomas and Harris Street, Ultimo NSW 2007. Email: Diane.McDougald@uts.edu.au

Singapore Centre for Environmental Life Sciences Engineering, Nanyang Technological University, Singapore, 60 Nanyang Drive, Singapore 637551. Email: rscott@ntu.edu.sg

**Supplementary information**

**Table S1:** PERMANOVA of factors on DNA based microbial community changes

|  | Df | SumsOfSqs | MeanSqs | F. Model | r^2^ | Pr(>F) |  |
| --- | --- | --- | --- | --- | --- | --- | --- |
| Counts | 1 | 0.09484 | 0.094837 | 33.145 | 0.27176 | 0.001 | *** |
| Phase | 2 | 0.12363 | 0.061814 | 21.604 | 0.35426 | 0.001 | *** |
| Reactor | 3 | 0.0132 | 0.0044 | 1.538 | 0.03782 | 0.184 |  |
| Residuals | 41 | 0.11731 | 0.002861 |  | 0.33616 |  |  |
| Total | 47 | 0.34898 |  |  | 1 |  |  |

Signif. codes: 0 ‘***’ 0.001 ‘**’ 0.01 ‘*’ 0.05 ‘.’ 0.1 ‘ ’ 1

Permutation: free, Number of permutations: 999

Call: adonis(formula = tgenus.bc2 ~ Counts + phase + reactor, data = samplesFile3, permutations = 999, parallel = 10)


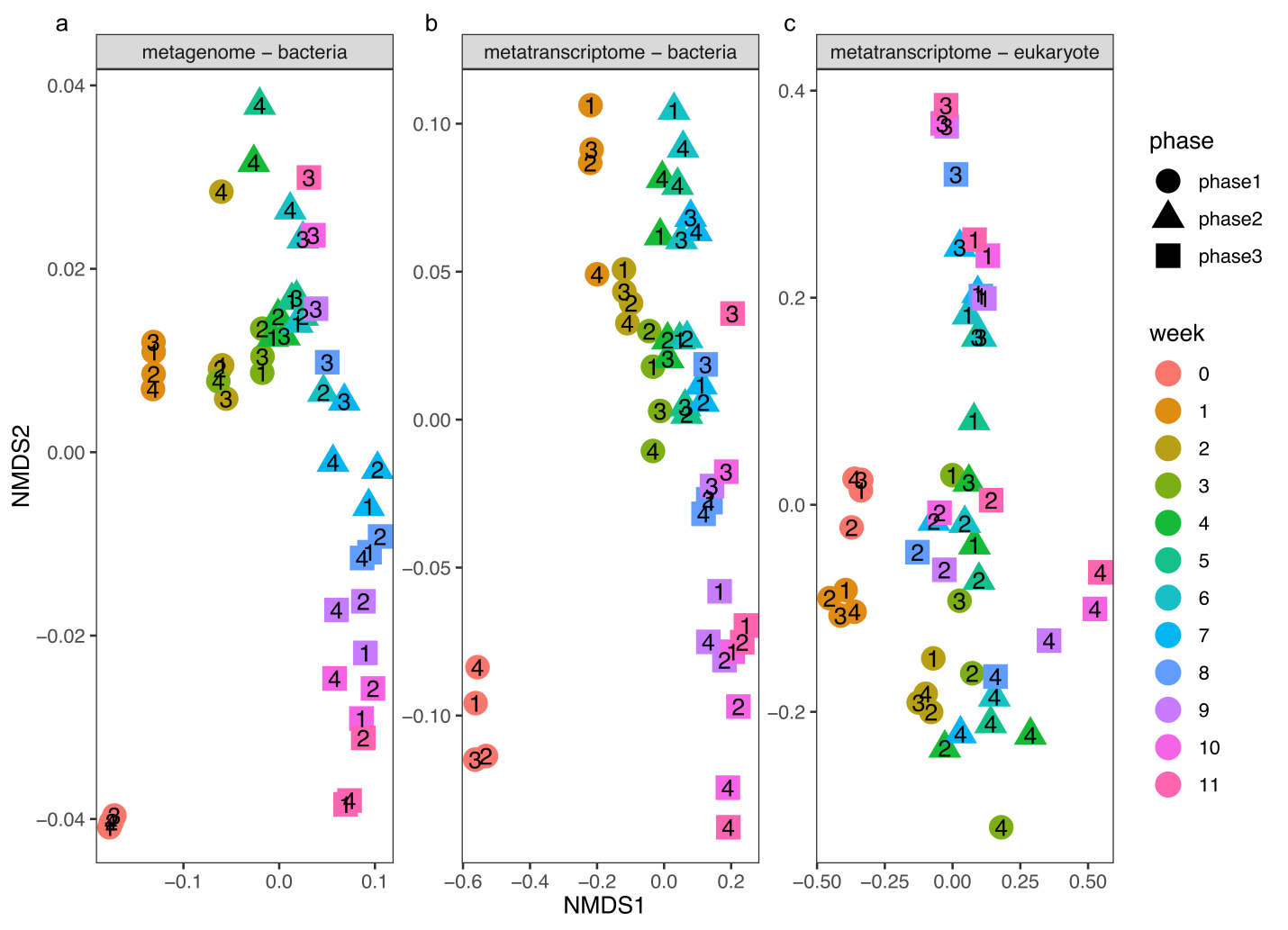


**Figure S1**: Non-metric multi-dimensional scaling (NMDS) plots for the microbial communities based on total metagenome (a) and total metatranscriptome (b, c) methods for 4 SBRs. The microbial abundance was ordinated using Bray-Curtis dissimilarity.


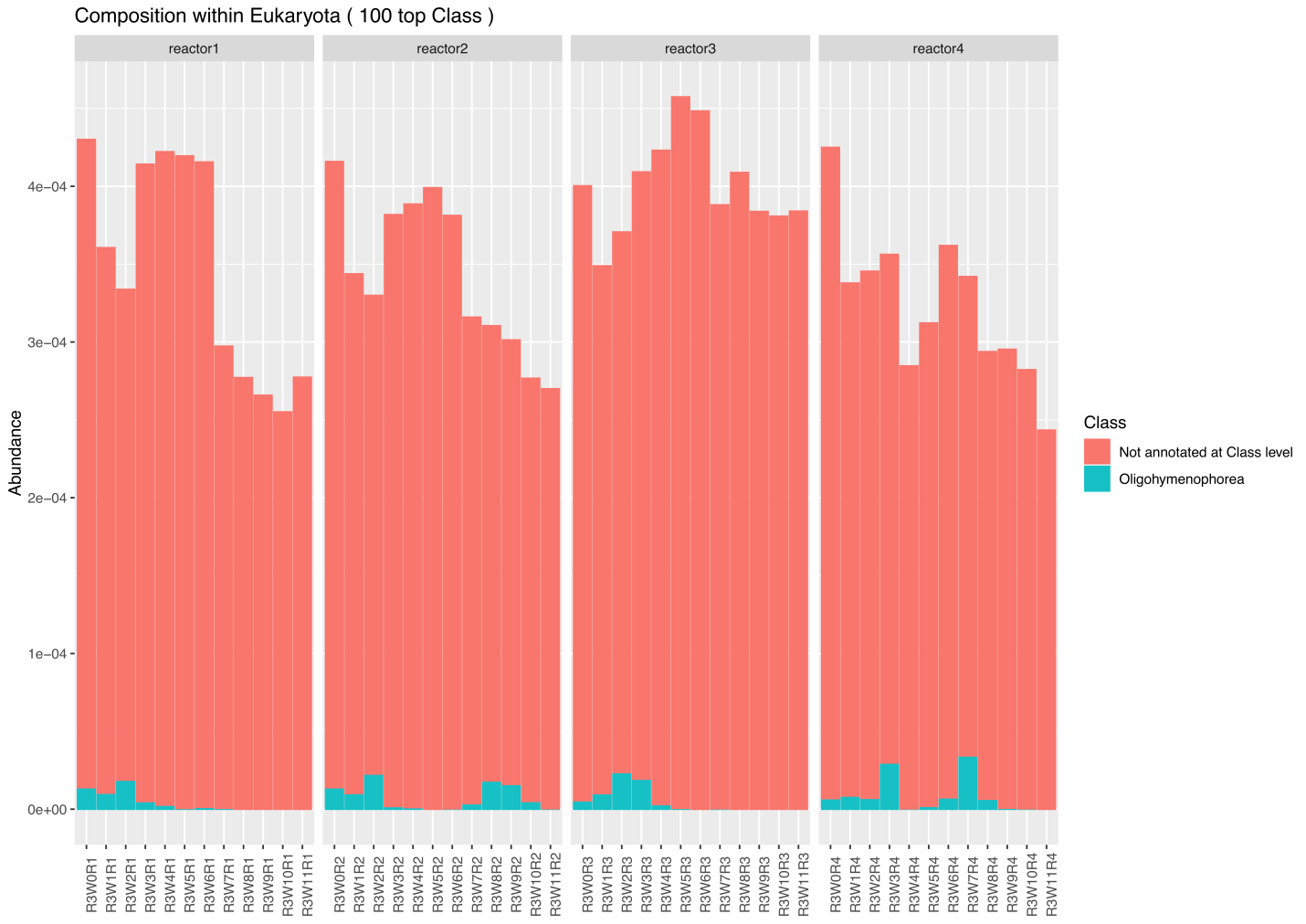


**Figure S2**: Relative abundance barplots of eukaryotic communities based on total metagenomic methods has a lack of annotation.


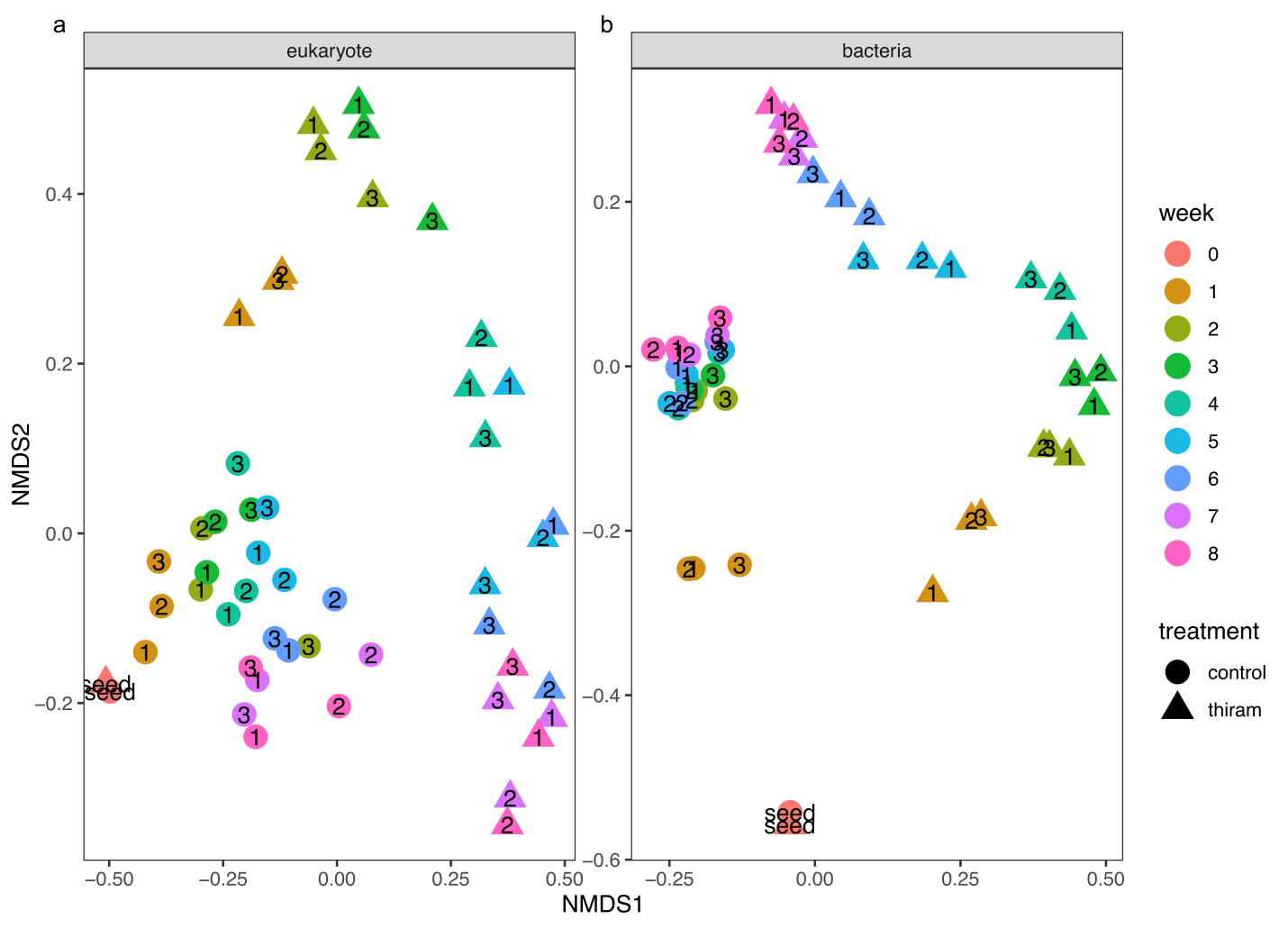


**Figure S3**: Non-metric multi-dimensional scaling (NMDS) plots for the (a) eukaryotic and (b) bacterial communities based on the total metatranscriptome method for control (●) and thiram-treated (▲) sludge in the mSBRs. The microbial abundance was ordinated using Bray-Curtis dissimilarity.

**Figure S4**: Abundance of microbial populations in the mSBRs over 8 weeks of operation. All reads counts are natural log transformed before analysis.
